# Isolation and Structural Characterization of Refined Proanthocyanidin Fractions Reveal Polymerization and Prodelphinidin Ratio as Major Contributors to Antiparasitic Activity

**DOI:** 10.1021/acsomega.6c01245

**Published:** 2026-08-21

**Authors:** Milla M. Leppä, Andrew R. Williams, Maarit Karonen, Juha-Pekka Salminen, Marica T. Engström

**Affiliations:** † Natural Chemistry Research Group, Department of Chemistry, 8058 University of Turku, Turku FI-20014, Finland; ‡ Department of Veterinary and Animal Sciences, Faculty of Health and Medical Sciences, University of Copenhagen, København DK-1165, Denmark; § Bioanalytical Laboratory, Institute of Biomedicine, Faculty of Medicine, University of Turku, Turku FI-20014, Finland

## Abstract

Proanthocyanidins are a highly bioactive group of plant-specialized metabolites. The aim of this study was to investigate the antiparasitic activity of a comprehensive set of highly purified and characterized proanthocyanidin fractions with versatile procyanidin/prodelphinidin ratios, mean degrees of polymerization, and galloyl contents. In total, 111 characterized fractions from 11 plant species were tested against the parasitic nematode *Ascaris suum* using migration inhibition and mortality assays. The results showed that a high mean degree of polymerization and a high proportion of prodelphinidins increased the antiparasitic activity of the fractions. The galloylated proanthocyanidin fractions did not possess especially high antiparasitic activity because of their low mean degree of polymerization. In general, highly procyanidin-rich fractions had low activity, yet there were significant differences in activity among fractions, which consisted mainly of A-type proanthocyanidins and those, which consisted mainly of B-type proanthocyanidins.

## Graphical abstract

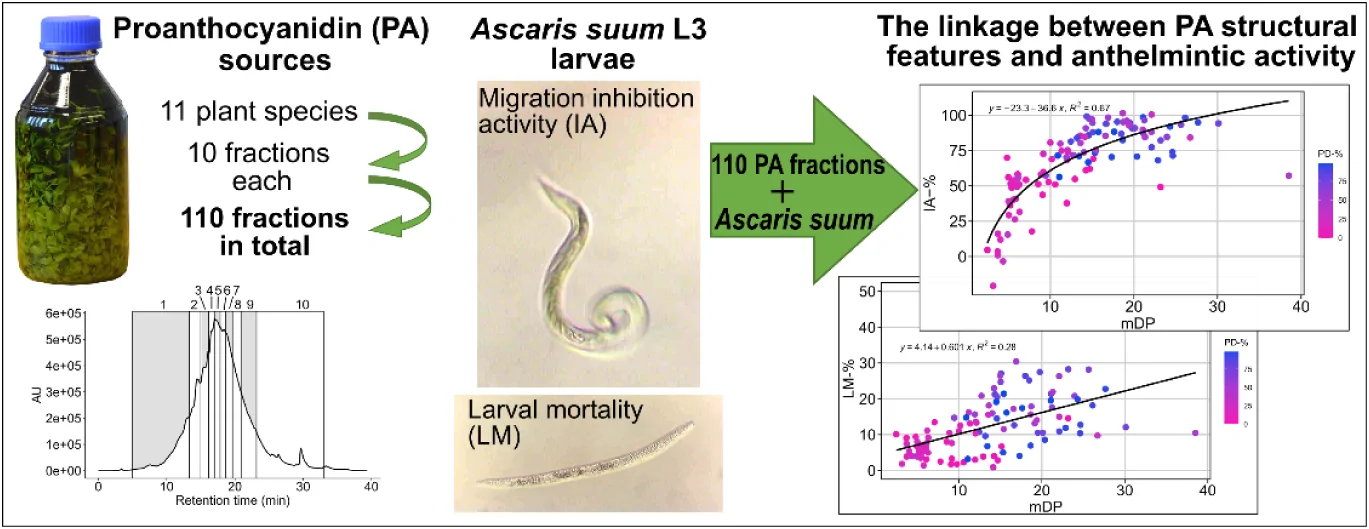

## Introduction

Proanthocyanidins (PAs) are one of the most potent plant-specialized metabolite groups present in terrestrial plants. They possess multiple different traits beneficial for animal^1^ and human health.^2^ In particular, PAs may offer a solution to several threats faced by modern-day agriculture.^3,4^ One of the most urgent threats is the continually increasing resistance of gastrointestinal nematodes (GINs) to synthetic anthelmintic drugs.^5,6^ Several studies have shown that PA-rich plant species can lower the GIN burden of ruminants,^7^ and self-medication behavior with PA-rich feeds has been observed in GIN-infected animals.^8,9^ Multiple studies have also shown the anthelmintic activity of PAs against GINs in vitro.^10–14^ PAs also possess anthelmintic activity against drug-resistant strains of nematodes, and these anthelmintic effects give rise to the possible use of PA-rich plants as nutraceuticals to suppress GIN burdens and improve animal health.^15^

PAs are a promising but challenging group of plant-specialized metabolites due to their complex structures. PAs consist of flavan-3-ol monomers, the most common of which are (epi)­catechin and (epi)­gallocatechin.^16^ Plants produce up to hundreds of individual PA molecules with varying polymer lengths and monomeric compositions.^17^ The molecular weights of PAs vary from hundreds of daltons to tens of thousands of daltons. The most common monomeric units are procyanidins (PCs) and prodelphinidins (PDs). Single PA oligomers or polymers cannot be efficiently separated from the naturally occurring mixtures via reversed-phase (RP) elution, and even the most refined fractions can contain tens of individual PA molecules.^17^ Thus, in many PA studies, the mean degree of polymerization (mDP) has been commonly used as a measure of the average polymer length of naturally occurring PA mixtures.

Previous studies have suggested that high polymer size increases the antiparasitic activity of PAs.^13,18,19^ Other beneficial structural features for the antiparasitic activity include the trihydroxylation of the flavan-3-ol B ring^13,19^ and the galloylation^18,20,21^ of the monomeric units. In contrast, the stereochemistry of the flavan-3-ols does not seem to have a strong effect on the activity.^18^

In most assays of antiparasitic activity, the utilized PA sources have been either plant tissues, extracts, or fractions.^11,14,19^ The variability of the PA structures has usually originated from the different plant sources or from crude fractionation of the PAs into mixtures of high- and low-mDP fractions.^19^ To our knowledge, the variation of the activity within a single PA mixture has not been studied in further detail. In this study, prepurified PA mixtures were each fractionated into ten subfractions of high purity and characterized by UHPLC-MS/MS and UPLC-DAD-Q-Orbitrap. The ability of the characterized fractions to inhibit migration and cause mortality in vitro against parasitic larvae of *Ascaris suum*, a highly prevalent swine parasite, was measured. This assay represents a convenient and highly reproducible in vitro system that can serve as a model system for many different parasitic nematodes.^22,23^ Utilization of this model allowed us to examine the structure–function relationship of the antiparasitic activity of PAs in unprecedented detail.

## Materials and Methods

### Solvents and Chemicals

Analytical-grade acetone and methanol (VWR International S.A.S., France) were used in the extraction and Sephadex LH-20 column chromatography. Semipreparative HPLC analyses were carried out with HPLC-grade acetonitrile (VWR International S.A.S., EC). Formic acid (99–100%, VWR Chemicals, EC) was used for eluent buffering. For the UHPLC analyses, LC-MS-grade acetonitrile was purchased from VWR International S.A.S. (USA) and LC-MS-grade formic acid from Sigma-Aldrich. The water used in the extraction, purification, and UHPLC-MS/MS and UHPLC-HRMS analyses was purified with A Millipore Synergy UV (Merck KGaA, Darmstadt, Germany) system.

### Isolation and Fractionation of Proanthocyanidins

The same plant species were utilized in this study as in Leppä et al.^17^ The selected PA-rich plant species functioned as a source of structurally variable PAs (high and low mDP, different PC/PD ratios, and possible galloylation). The selected plant species, plant parts, and their PA compositions are presented in the Supporting Information (Table S1). The protocols used for extraction and PA isolation were also similar to those in the aforementioned paper.^17^ Shortly, plants were freshly collected into acetone and macerated for 9–12 months at +4 °C. Plant material was ground and extracted with 4/1 acetone/water (v/v) 5–7 times. Extracts were brought to the water-phase and freeze-dried. Lyophilized extracts (8–10 g) were dissolved in a small volume of water (40–50 mL) and insoluble impurities were discarded prior to Sephadex LH-20 column chromatography (40 × 4.0 cm, Chromaflex, Kontes). The column was flushed gradually with water, methanol/water (3/7 and 1/1, v/v), and acetone/water (3/7, 1/1, and 4/1, v/v), and the PA-rich fractions were collected with acetone/water (4/1, v/v). Organic solvents were evaporated and the collected fractions were freeze-dried.

Also, the next purification step was performed as described by Leppä et al.^17^ Lyophilized PA-rich Sephadex LH-20 fractions were further separated with an HPLC system equipped with a semipreparative column (150 × 21.20 mm, Gemini 10 μm, C-18, 110A, Axia packed, Phenomenex). Shortly, 125–150 mg of Sephadex LH-20 fraction in water was injected into the system, and the elution was carried out at a flow rate of 12 mL min^–1^ and two eluents—A: acetonitrile and B: formic acid/water, (0.1/99.9, v/v)—were used. The gradient elution started with isocratic 8% A in B for 0–4 min, followed by a linear gradient of 8–55% A in B for 4–32 min. The column was washed with 80% A in B and stabilized with 8% A in B prior to the next analysis.

Fractions were collected into 2 mL tubes in a 28-min time frame starting from either 5, 7, 8, or 10 min depending on the retention time of the eluted PAs (Supporting Information Figures S1 and S2). In total, 168 fractions were collected per sample. First, the UV chromatograms (λ = 280 nm) of samples were integrated from the collected 28-min retention-time frame with Target Lynx software (V4.1 SCN876 SCN 917 2012 Waters Inc.). The resulting total UV area was divided into 10 equal parts, and new integrations for the same retention-time area were made with preset values, thus dividing the collected area into 10 “slices” (Supporting Information Figures S1 and S2). The collected 168 fractions were then pooled according to the retention times obtained from the second integration. In total, 10 pooled semipreparative fractions were obtained for each plant sample. Pooled fractions were brought to the water phase in vacuo, after which they were freeze-dried. All pooled fractions were analyzed by UHPLC-DAD-MS/MS and UHPLC-DAD-Q-Orbitrap as described below.

### UHPLC-DAD-MS/MS Analyses

The UHPLC-DAD-MS/MS analyses^24,26^ were carried out with a Xevo TQ triple quadrupole mass spectrometer (Waters Corp., Milford, MA, USA), which was coupled to an Acquity UPLC system (Waters Corp., Milford, MA, USA). The column used was a Waters Acquity UPLC BEH Phenyl (1.7 μm, 2.1 × 100 mm; Waters Corp., Wexford, Ireland). For the elution, acetonitrile (A) and 0.1% aqueous formic acid (B) were used at a flow rate of 0.5 mL min^–1^. Two elution profiles were utilized in the analysis of the samples. The elution profile of 9.5 min was used for the earlier-eluting PA fractions, whereas the profile of 11.5 min was used for the late-eluting PA fractions. The 9.5 min elution profile was as follows: 0–0.5 min, 0.1% A in B (isocratic); 0.5–5.0 min, 0.1–30% A in B (linear gradient); 5.0–6.0 min, 30–35% A in B (linear gradient); 6.0–9.5 min, column wash and stabilization. In the 11.5 min elution, the beginning of the elution was similar to the aforementioned gradient, but the latter linear gradient lasted until 8 min, changing the rest of the elution profile as follows: 5.0–8.0 min, 30–45% A in B (linear gradient); 8.0–11.5 min, column wash and stabilization. The data were recorded from 0 to 6 min with the 9.5 min gradient and from 0 to 8 min with the 11.5 min gradient. The ion source and detection parameters at the DAD and MS/MS were similar to those in previous studies utilizing the Engström method.^17,24–26^ The stability of the ionization and MS conditions was monitored with frequent injections of a 1 μg mL^–1^ catechin solution (in acetonitrile/0.1% formic acid (2/8, v/v)) throughout the analysis cycle.^25^

The detection of the PC and PD units, as well as galloyl groups, was carried out with the Engström method.^24,26^ The traces were quantified as described in previous studies.^17,24–26^ In short, the PC and PD traces were smoothed (window size 5 scans × 2 smoothing iterations) prior to integration, and the integrations were performed with Target Lynx software (V4.1 SCN876 SCN 917 2012 Waters Inc.). The integrated areas were converted into concentrations based on calibration curves that were obtained earlier.^24,26^ The galloyls were quantified against a dilution series of 1,2,3,4,6-penta-*O*-galloylglucose, and Sephadex LH-20 fractions, whose PC/PD contents and mDP had previously been quantified against thiolysis results, were used to produce calibration curves for the PC and PD units and mDP.^24^ The estimation of the galloyl content in relation to PA content (EG, mol/mol) was calculated based on the quantitative results of the galloyls^26^ and PAs.^24^

### UHPLC-DAD-HRMS Analyses

The high-resolution mass spectra (HRMS) were measured in a similar way as described by Leppä et al.^17^ In short, the analyses were carried out with a quadrupole–Orbitrap instrument (QExactiveTM, Thermo Fisher Scientific GmbH, Bremen, Germany), which was coupled to a similar UHPLC system as described above (Acquity UPLC, Waters Corp., Milford, MA, USA). The samples were analyzed with an Acquity UPLC BEH Phenyl 1.7 μm, 2.1 × 30 mm column, and the analytes were brought to the gas phase via a heated ESI source (H-ESI II, Thermo Fisher Scientific GmbH). The reversed-phase elution was carried out with (A) acetonitrile and (B) formic acid/water (0.1/99.9, v/v), and the flow rate was set to 0.65 mL min^–1^. The elution profile was as follows: 0–0.1 min isocratic 3% A in B, 0.1–3.0 min linear gradient 3–45% A in B, and last 3.0–4.2 min column wash and stabilization. The UV (λ = 190–500 nm) and mass (*m*/*z* = 200–3000, resolution = 70 000, automatic gain control = 3 × 10^6^) data were recorded throughout the analysis, and the analytes were detected as negative ions. Full-scan mass spectra were interpreted with Qual Browser in Thermo Xcalibur (version 4.1) software.

### Larval Migration Inhibition and Mortality Assay

The *Ascaris suum* stage L3 larvae were prepared from previously collected embryonated *Ascaris suum* eggs,^27^ which were decoated with NaOH and stored at +4 °C in 1 M H_2_SO_4_ as described by Williams et al.^13^ The eggs were thoroughly washed with Hank’s Balanced Salt Solution (HBSS) by centrifugation at 500 g, replacing the supernatant 3–4 times. The washed eggs were gently ground with glass beads in HBSS at +37 °C, 5% CO_2_ in air for 40–60 min. The hatched larvae were separated from debris by allowing the larvae to migrate into sterile HBSS through a 20 μm sieve overnight at +37 °C, 5% CO_2_ in air.

The migration inhibition assay used in this study was modified from the previously described approach by Williams et al.^13^ First, *Ascaris suum* stage L3 larval concentration was set to 0.67 μL^–1^ in cell culture medium (RPMI 1640 supplemented with 2 mM l-glutamine, 100 U/mL penicillin, and 100 μg mL^–1^ streptomycin), and 145 μL of larval solution was dispensed into a 96 well cell culture plate. The tannin samples were dissolved in DMSO/water for injection (WFI, 1/1, v/v), and the concentration of the tannin solutions was adjusted to 3.0 mmol mL^–1^. Samples were added to the wells by pipetting 5 μL of sample into the larval suspension. The plates were incubated overnight at 37 °C in 5% CO_2_ in air. PA and control samples were analyzed in eight replicates. Each plate contained a total of nine PA samples, as well as positive (50 μg/mL ivermectin) and negative (DMSO/WFI (1/1, v/v)) controls.

After incubation, the numbers of dead and living larvae per each well was counted under an inverted light microscope. Next, 150 μL of 1.6% agar solution in water (45 °C) was added to each well, and the solutions were mixed thoroughly. At the same time, half of the agar mix was transferred into a fresh cell culture plate, resulting in two identical plates, both containing 150 μL of agar-cell culture medium mix in each well. The agar was allowed to solidify, and 100 μL of fresh medium was added into the wells. Plates were incubated overnight under the same conditions as previously. The number of migrated larvae was counted on top of the set agar using a normal light microscope. The larval counts of the identical plates were combined before result calculation.

Larval migration inhibition activity percentages (IA%) were calculated relative to the maximal and minimal migration percentages obtained from negative and positive control samples, respectively. The larval mortality percentage (LM%) was calculated in a similar manner. The error bars in the bar plot (Figure 4) are presented as the standard error of the mean.

## Results and Discussion

### The Effects of Proanthocyanidin Structural Features on Antiparasitic Activity across Plant Species

Figure 1 shows the effect of mDP and PD content on the measured antiparasitic activity across all the studied PA fractions. The plant-species-specific IA% and LM% as a function of mDP are presented in the Supporting Information (Figure S3). As seen in Figure 1, both the migration inhibition activity and larval mortality increased as a function of the mDP. The IA% increases steadily until a certain mDP is reached, after which the antiparasitic activity levels off. The increase in inhibition followed a logarithmic curve (*y* = −23.3 + 36.6 × ln­(*x*), *R* ^2^ = 0.67) and was mostly caused by the saturation of the IA% of the complete data set at high polymer size (approximately mDP > 16, Figure 1A). However, Figure 1A also shows that when the mDP is increased over a certain value, the antiparasitic activity decreases, most drastically in the fraction with an mDP of 38 (Figure 1A). This may be due to steric hindrance and/or stronger self-association, which may prevent at least some mode of action. Although the antiparasitic effects of PAs are not yet fully understood, it has been suggested that these are linked to their protein-binding properties, and disturbance of key enzyme functions is one potential mode of action.^28^ In addition, the coating of the parasite surface interferes with the function of key parasite proteins and the free movement of larvae.^13^ Interestingly, previous studies have shown that tannin size and protein-binding capacity also have an upper limit, as the steric hindrance of large tannins.^29,30^

**Figure 1:**
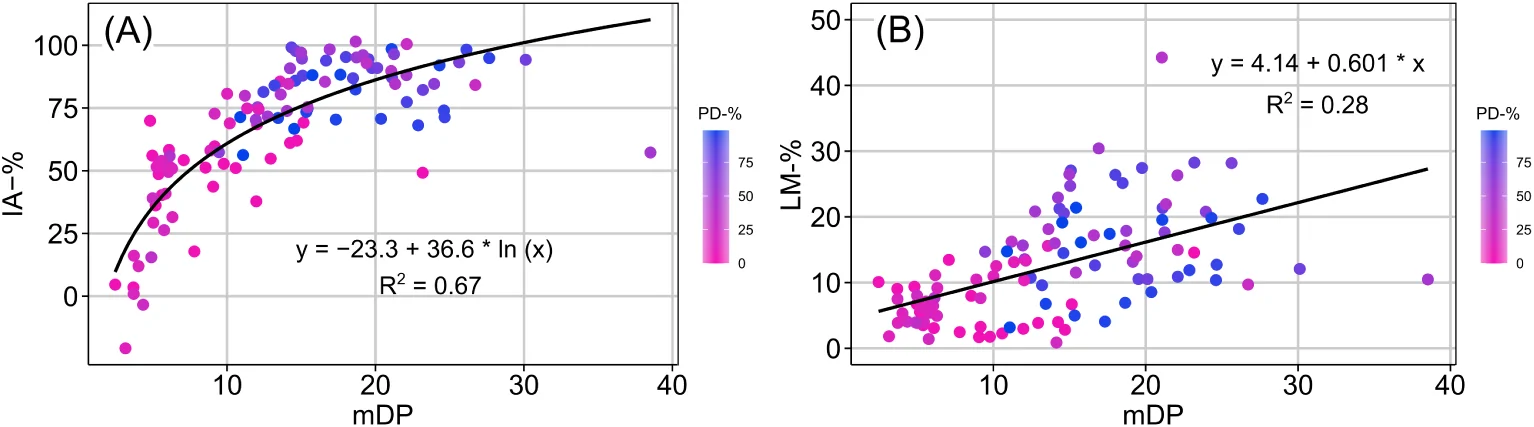
(A) Migration inhibition activity (IA%) and (B) larval mortality (LM%) of the studied proanthocyanidin samples as a function of the mean degree of polymerization (mDP). The proportion of prodelphinidins (PD%) in relation to total proanthocyanidins is illustrated as a color gradient (PD%: blue = 100%, pink = 0%).

In general, the least-inhibitive fractions (IA% < 50%) contained PC-rich low-mDP (<10) PAs, while the most-inhibitive fractions (IA% > 75%) contained PD-rich high-mDP (>10) PAs. Also, the mortality increased as the mDP increased (Figure 1B), yet the pattern was not as clear as it was with the IA%. The LM% followed a linear ascending curve as a function of mDP (*y* = 4.41 + 0.601 × *x*, *R* ^2^ = 0.28). However, for both IA% and LM% the most active fractions mortality-wise, were slightly PD-rich PAs (PD% ≥50%), while the least active fractions were low in mDP and high in PC units. In general, the LM% values were much lower than the IA% values, which is intuitive as migratory inhibitory activity is inherently sublethal and will be manifested at lower PA concentrations. In the present study, the concentrations used were expected to be physiologically relevant, as they are likely to occur in the gastrointestinal tract of animals following a moderate intake of PA-rich feedstuffs (see discussion in Williams et al.^13^). The LM% of the samples ranged between 0% and 30%, except for a single outlier (LM% = 44%).

Altogether, the observed increased antiparasitic activity of high-mDP and high PD% fractions is in good accordance with the previous literature.^13,18,19^ Although in this case the mDP and PD% had high collinearity, and thus their relevance to the activities cannot be necessarily distinguished. Nevertheless, probably both structural features were significant, since trihydroxylation of flavan-3-ols and high mDP have been associated with increased antiparasitic activity.^1,18^

### The Effect of the Retention Time of the Fractions on Antiparasitic Activity

The main factors defining the elution order of PAs in a reversed-phase (RP) system are their stereochemistry and polarity.^31–33^ Particularly, trihydroxylation of the B-ring (PDs) enhances the polarity of flavan-3-ols, thus reducing their retention in the RP column.^32^ The structural complexity of plant PAs affects their retention profiles in RP-LC, resulting in the characteristic unresolved chromatographic humps. Therefore, we wanted to explore if the antiparasitic activity could be linked to the chromatographic behavior of the PA fractions and thus, certain parts of the chromatographic profiles of PAs in general. The distribution of the two antiparasitic activities as a function of retention time is presented in Figure 2. The size of the bubbles represents the mDP of the individual fractions, and the gradient color from blue to pink illustrates the proportion of PD units (PD%) in the complete PA fingerprint of each fraction. From the IA% point of view (Figure 2A), the majority of the PD-rich and relatively active fractions (IA % >50%) had relatively early retention times (tR = 2.2–4.0 min, Figure 2A), while the least active (IA% <50%), low-mDP (≤10), PC-rich fractions had later retention times (tR = 3.3–5.5 min). The most active fractions (IA% > 90%) were widely distributed across the observed retention time range (tR = 2.8–5.3 min) and included both PD-rich fractions and fractions with mixtures of PC and PD units.

**Figure 2:**
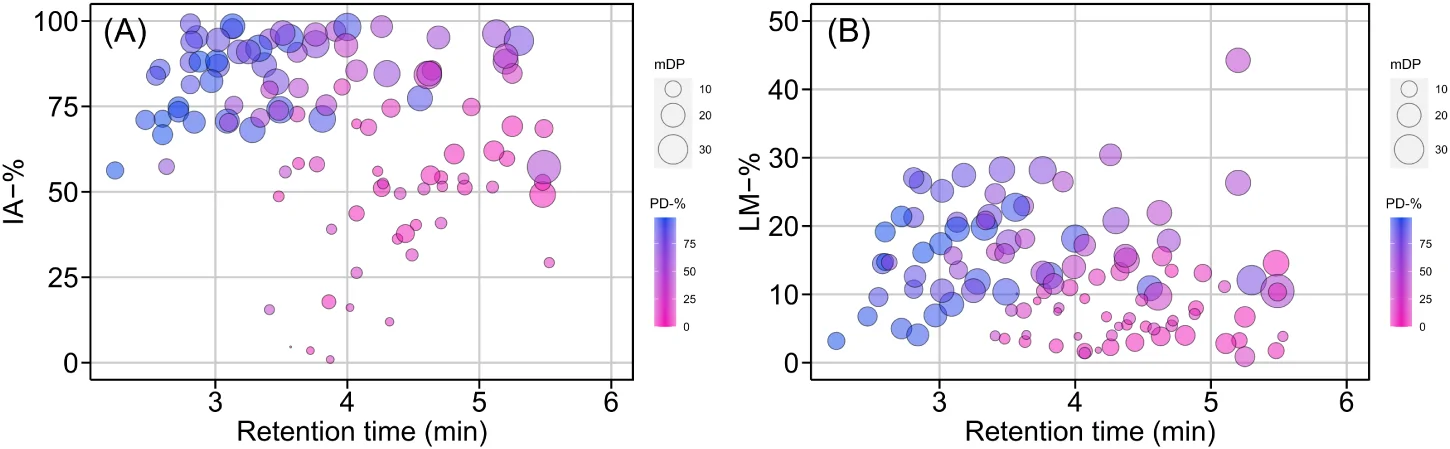
(A) Migration inhibition activity (IA%) and (B) larval mortality (LM%) of the studied proanthocyanidin samples as a function of the retention time of the fractions. The proportion of prodelphinidins (PD%) in relation to total proanthocyanidins is illustrated with a color gradient (PD%: blue = 100%, pink = 0%). The size of the bubbles illustrates the mean degree of polymerization (mDP) of the fractions.

The distribution of LM% is illustrated in Figure 2B. Naturally, the distribution of the PC and PD units remained similar to those in the case of IA% (Figure 2A), meaning that the PD-rich fractions eluted at early retention times (tR = 2.2–4.0 min, Figure 2B) and the PC-rich ones at later retention times (tR = 3.3–5.5 min). Since the LM% values were rather low, differences in the antiparasitic activity were less evident than in IA%. Nevertheless, all the late-eluting (tR = 3.3–5.5 min), low mDP (≤10), PC-rich fractions showed low LM% (≤15%). It also seemed that the early-eluting PAs (tR < 3.3 min) were at least moderately active in the inhibition of larval migration.

These findings showed that there was not a single time point in the PA fingerprints that was significantly more active than any other retention time window. This is not that surprising, as the distribution of PAs across the retention time window is affected by the same features that also affect their antiparasitic activities. However, while increasing the mDP and PD content increases the antiparasitic activity of PAs, the former increases the retention times and the latter shortens the retention times in RP-LC. In addition, in the present study, the saturation of the IA% with the PA concentration used (100 mM) partially disabled finding retention time differences among the most active fractions. More variation and nuances in the IA% would have been observed if more diluted PA solutions had been used for the most active plant species.

### Antiparasitic Activity within and between Plant Species

To better understand the antiparasitic activities of the studied fractions, we next compared them within and between different plant species. Figure 3 shows how the antiparasitic activities of the fractions from individual plant species were located in Figure 1 as a function of mDP. In addition, the IA% and LM% results of each plant species, fraction-by-fraction, are presented Figure 4. When considering all ten fractions from each plant species, the most inhibitory PA fractions were from *P. sylvestris*, *S. phylicifolia*, *L. vulgaris*, and *T. repens* (Figure 3A, Figure 4). The fractions originating from these plants were also active in terms of mortality, although *P. sylvestris* and *S. phylicifolia* had higher LM% values than *L. vulgaris* and *T. repens* (Figure 3B). A similar difference in the migration inhibition activity might also have been observed if the PA concentration had been lower. As the response of the IA% saturated at approximately mDP < 16, the differences between the fractions of *L. vulgaris*, *P. sylvestris*, *S. phylicifolia*, and *T. repens* diminished (Figure 4G-I, K). The fractions of *T. repens* were extremely PD% rich (PD% = 76–99%), while the variation in PD% was greater in *P. sylvestris* (PD% = 47–85%), *S. phylicifolia* (PD% = 8–93%), and *L. vulgaris* (PD% = 30–92%). The mDP of the fractions varied between 11 and 39. Overall, the most active fractions were high in both mDP and PD%.

**Figure 3:**
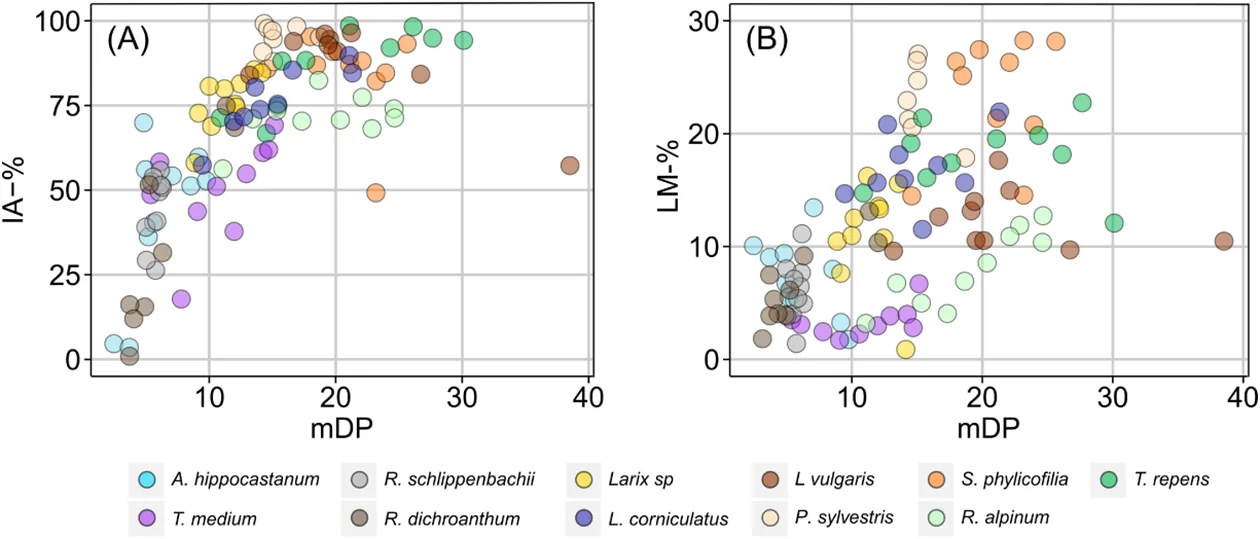
(A) Migration inhibition activity (IA%) and (B) larval mortality (LM%) of the studied samples as a function of the mean degree of polymerization (mDP). The plant species are illustrated with different colors.

**Figure 4:**
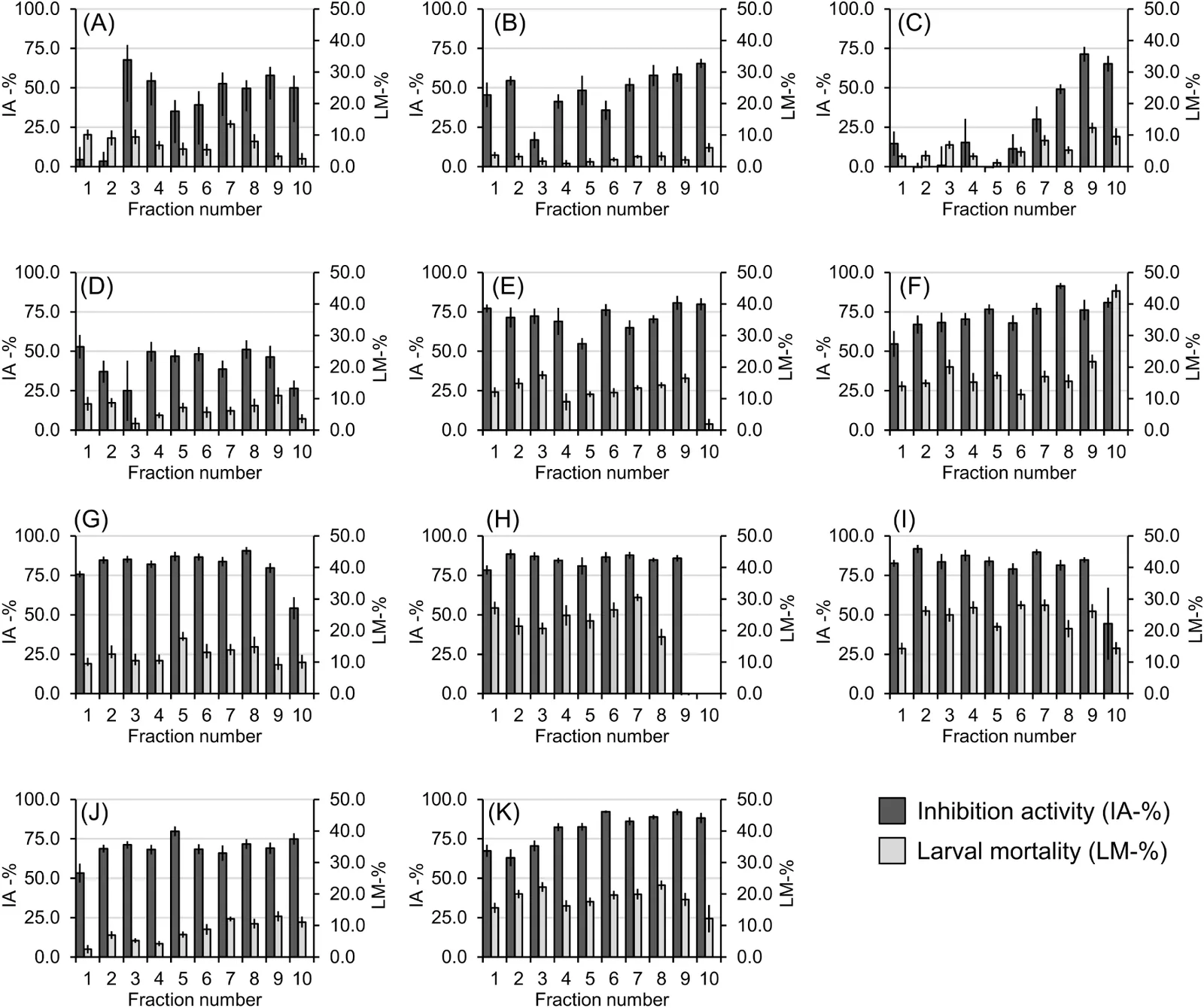
Migration inhibition activity (IA%) and larval mortality (LM%) of each studied fraction of all plant species. Dark gray: inhibition activity % (IA%), light gray: LM%, (A) *Aesculus hippocastanum*, (B) *Trifolium medium*, (C) *Rhododendron dichroanthum*, (D) *Rhododendron schlippenbachii*, (E) *Larix* sp, (F) *Lotus corniculatus*, (G) *Lysimachia vulgaris*, (H) *Pinus sylvestris*, (I) *Salix phylicofilia*, (J) *Ribes alpinum*, and (K) *Trifolium repens*.

Even though high PD% indicated high antiparasitic activity, particularly high IA%, the activity was not similar in both extremely PD-rich plant species of this study. For instance, *R. alpinum* (Figure 4J) fractions were high in mDP (>15) and PD % (>90%), yet their activity was lower (IA% < 75%) compared to the fractions of *T. repens* (IA% = 67–99%, Figure 4K) with the same PA composition (Figure 3A). These samples were analyzed by high-resolution mass spectrometry for further details (Supporting Information Figure S4 and S5) but they did not show clear differences between the plant species. PA fractions from both plant species consisted of highly PD-rich oligomers and polymers, and their degree of polymerization ranged from 4 to 19. However, the comparison of the UV traces (λ = 280 nm, Supporting Information Figure S6) of *R. alpinum* and *T. repens* fractions showed that the retention time windows of the fractions of *R. alpinum* were wider than those of *T. repens*. The elution behavior of PAs in RP system is mainly affected by the stereochemistry and polarity of the compounds.^31–33^ For instance, epigallocatechins elute at later retention times than gallocatechins^32^ and PAs with B-type linkage C4–C6 elute later than those with C4–C8 linkage type.^33^ These kinds of structural features could cause the observed differences between the elution profiles of *R. alpinum* and *T. Repens* and, in addition, cause the differences in their antiparasitic activities.

A few plant species had fractions with medium to high antiparasitic activity (Figure 3). Such plant species were *Larix sp*. and *L. corniculatus*, and their activities were IA% = 58–86% and IA% = 57–100%, as well as LM% = 1–16% and LM% = 12–44%, respectively. The PA fractions from both plant species were mixtures of PC and PD units, and their PD% was 12–90% and 41–65%, respectively. The mDP of the fractions of *Larix sp*. varied less (mDP = 9–14) than the mDP of *L. corniculatus* fractions (mDP = 10–21). Especially in these plant species, the increase in the IA% followed a linear curve as a function of the mDP (*Larix sp*., *R* ^2^ = 0.583 and *L. corniculatus*, *R* ^2^ = 0.693; Figure S3E, F, respectively).

The plant-by-plant comparison revealed that the least active PA fractions in this study were from plant species *A. hippocastanum*, *T. medium*, *R. dichroanthum*, and *R. schlippenbachii* (Figures 3 and 4A–D). Both *R. dichroanthum* and *R. schlippenbachii* contained galloylated PAs, while *T. medium* and *A. hippocastanum* contained nearly PC-pure PAs (PD% = 1% and 1%, respectively). Especially, the inhibitory activities of these fractions were lower (IA% <75%, Figure 3A) than those of the rest of the plant species. Also, the mortalities (LM% <15%, Figure 3B) of these fractions were low. The low activity of these fractions is explained by their low mDP (<10, Figure 3A) in comparison to the rest of the analyzed fractions.

Previous studies have shown that the galloylation of flavan-3-ols increases their antiparasitic activity in comparison to nongalloylated ones,^18–20,34^ An increased activity of galloylated PAs was not observed in the present study, most likely due to the low mDP of the galloylated PA fractions as well as the low galloyl content of these fractions. The estimated galloyl content in relation to PA content (EG) of *R. dichroanthum* and *R. schlippenbachii* fractions was <1 (mol/mol) in the majority of the fractions (Figure S7). Also, no correlation between EG and either IA% or LM% was observed (Figure S7). The PD% of the galloylated PAs was relatively low (PD% = 10–40%; Figure S8), which could also be a partial reason for the low activity.

The results of the most PC-rich plant species of this study, *A. hippocastanum* and *T. medium*, showed the relevance of other structural features of PAs besides mDP. The fractions of *T. medium* had very low LM% (=2–7%) even though their mDP (= 4–16) was relatively high. On the other hand, the fractions of *A. hippocastanum* had higher LM% (= 2–14) even though their mDP (= 2–10) was lower than those of *T. medium* (Figure 3B). The PD% of the individual fractions of these plant species were similar to each other as well (*A. hippocastanum*: PD% = 0.8–2.1%, *T. medium*: PD% = 1.3–3.5%), meaning that the difference in activity must originate from some other structural feature than the polymer size or PC/PD composition. The more detailed examination of *A. hippocastanum* and *T. medium* via HRMS (Supporting Information Figures S9 and S10) showed compound-specific differences in the compositions of the aforementioned plant species. As presumed based on the MS/MS data, both plant species contained mainly extremely PC-rich PA oligomers and polymers. The comparison of the HRMS data showed that the *A. hippocastanum* fractions contained mainly A-type PCs while, the PC oligomers and polymers in *T. medium* fractions were mainly B-type, even though *T. medium* fractions with later retention times (Supporting Information Figure S10C) contained A-type PAs to some extent. In addition, *A. hippocastanum* contained fewer individual compounds as compared to *T. medium* and the fractions of *T. medium* contained larger PA molecules. The UV chromatograms (λ = 280 nm) of the fractions of these plant species (Supporting Information Figure S11) supported these findings. The chromatograms of *A. hippocastanum* (Figure S11A) showed sharper peaks and narrower retention-time windows (tR = 0.45–3.0 min) of the actual PA fingerprints, while sharp peaks were present only in a few fractions of *T. medium*, and the retention-time windows (tR = 1.8–3.0 min) were much greater.

The probable reason for the higher antiparasitic activity of *A. hippocastanum* fractions as compared with *T. medium* is the substantially higher number of A-type PC oligomers in *A. hippocastanum* fractions. The A-type linkage between the flavan-3-ol units reduces their flexibility and solubility in water, thus increasing their hydrophobicity.^35^ The increased hydrophobicity alters their interaction with proteins and can lead to increased protein precipitation in A-type PAs.^36^ Previous studies have shown that similar structural features of PAs have been linked to both elevated antiparasitic activity as well as protein affinity and precipitation. This has led to the hypothesis that the antiparasitic activity of PAs is caused by their interaction with proteins.^12^ Our findings about the increased activity of PAs with A-type linkages and high mDP hereby support this hypothesis.

It should also be considered that PAs may not necessarily remain in their native forms under the assay conditions. In aqueous solutions, especially at pH values above 6.5, pyrogallol-type B-rings present in PDs are prone to oxidation.^37,38^ This autoxidation can generate reactive oxygen species such as hydrogen peroxide, which may contribute to antiparasitic effects^37^ and might partially explain the increased antiparasitic activity of PD-type PAs. In addition, the oxidation process can produce quinone intermediates that participate in coupling reactions with A-ring methine carbons, leading to oligomerization products.^38,39^ Such oxidative transformations have also been suggested to promote the formation of A-type interflavan linkages in PAs.^38,40^ Both types of modifications could contribute to the observed antiparasitic activity. Therefore, partial oxidation or structural modification of PAs during the assay cannot be excluded and may partly influence the biological activity observed.^41^

Additionally, this study did not address potential synergistic or antagonistic interactions between PAs and other molecules. Previous work has demonstrated synergistic effects between PAs and other specialized metabolites (e.g., refs 34,42 ). Interactions between PAs and commonly used anthelmintics have also been reported,^43–45^ alongside well-established interactions between PAs and other macromolecules.^46–49^ Future studies should therefore explore combinations of fractions with differing characteristics to assess potential synergistic effects, particularly as these compounds may operate through different modes of action. Also, one limitation of the present study is that these findings are derived from in vitro experiments. In vivo conditions within the intestine may differ substantially. Furthermore, although *A. suum* serves as a convenient and robust model nematode for structure–function studies, it cannot be assumed that these results are directly applicable to other nematode species, such as *H. contortus*. Ultimately, the efficacy of the observed effects should be determined empirically for each specific host–parasite system.

Current hypotheses on the antiparasitic mechanisms of PAs in nematodes emphasize their ability to bind to proteins or lipids on the external cuticle of the parasite. This interaction is thought to cause steric hindrance of feeding and movement, ultimately leading to starvation and death.^50^ The observed positive correlation between mDP and antiparasitic activity supports this hypothesis, as larger PAs may form more extensive PA–protein aggregates on the worm surface, thereby more rapidly inhibiting motility. Furthermore, the enhanced activity observed in PD-rich fractions suggests that an increased capacity for hydrogen bonding with parasite molecules, attributable to the additional hydroxyl group present in PD-type flavan-3-ols, results in stronger PA binding and, consequently, greater antiparasitic efficacy.

## Conclusions

In conclusion, a high mDP appears to be the key structural feature underlying antiparasitic activity. This is likely driven by strong interactions between PAs and parasite surface components, leading to the formation of aggregates on the worm cuticle that inhibit motility. However, further biochemical studies are required to confirm this mechanism of action. The high mDP did not always guarantee very high activity, as was evident in the comparison of *R. alpinum* and *T. repens*, where both plant species had similar mDP and PD%, yet their activities differed significantly. The most PC-pure fractions were the least active ones, but a high composition of A-type PC oligomers in *A. hippocastanum* increased the activity substantially as compared to highly PC-rich *T. medium*, which mainly consisted of B-type PC oligomers and polymers. The galloylated plant species *R. dichroanthum* and *R. schlippenbachii* did not seem to have higher activity than nongalloylated plants, but this was likely due to the low mDP and galloyl content of the studied fractions. To continue to unravel the antiparasitic effects of PAs, it would be important to investigate the stabilities of the PA fractions under physiological conditions and the possible modified products formed in such conditions. This would help to link the observed in vitro activities to in vivo studies and further increase the understanding of the mechanisms behind the anthelmintic activities of PAs. Consequently, this would facilitate their potential use in various tannin-based applications. The routine use of synthetic anthelmintic drugs in livestock agriculture is increasingly discouraged due to the ecotoxicity of drug residues, which may adversely affect soil microfauna, as well as the persistent risk of developing anthelmintic resistance. The present findings, which elucidate structure–function relationships between PAs and antiparasitic activity, may help guide the rational development of novel feed additives aimed at suppressing gastrointestinal parasite burdens. Such approaches could reduce infection levels below thresholds associated with significant production losses. In this way, synthetic anthelmintics could be reserved primarily for the therapeutic treatment of clinical disease outbreaks, thereby helping to maintain their long-term efficacy.

## Supplementary Material



## Notes

The plant species and tissues used in this study (Table S1). The UV chromatograms (λ = 280 nm) of the semipreparative fractionations (Figures S1 and S2). The migration inhibition activity (IA%) and larval mortality (LM%) of each studied fraction as a function of mean degree of polymerization (Figure S3). The high-resolution mass spectra of *T. repens* pooled semipreparative fractions (Figure S4). The high-resolution mass spectra of *R. alpinum* pooled semipreparative fractions (Figure S5). The UPLC UV traces (λ = 280 nm) of the pooled semipreparative HPLC fractions of *R. alpinum* and *T. repens* (Figure S6). The larval mortality (LM%) and migration inhibition activity (IA%) as a function of the estimated relative galloyl content (EG) of the galloylated PAs in the plant species *R. dichroanthum* and *R. schlippenbachii* (Figure S7). Migration inhibition activity (IA%) and (B) larval mortality (mortality%) of the studied samples as a function of the proportion of prodelphinidin units (PD%) in relation to procyanidins. (Figure S8). The high-resolution mass spectra of *A. hippocastanum* pooled semipreparative fractions (Figure S9). The high-resolution mass spectra of *T. medium* pooled semipreparative fractions (Figure S10). The UPLC DAD traces (λ = 280 nm) of the pooled semipreparative HPLC fractions of (A) *A. hippocastanum* and (B) *T. medium* (Figure S11)­(PDF)

## Abbreviations

ESI: electrospray ionization
HRMS: high-resolution mass spectra
IA%: larval migration inhibition activity percentages
LM%: larval mortality percentage
mDP: mean degree of polymerization
MS: mass spectrometry
MS/MS: tandem mass spectrometry
MW: molecular weight
PA: proanthocyanidin
PC: procyanidin
PD: prodelphinidin
RP: reverse phase
UHPL,: ultrahigh-performance chromatography
