# Supplementary material for: Isolation and Structural Characterization of Refined Proanthocyanidin Fractions Reveal Polymerization and Prodelphinidin Ratio as Major Contributors to Antiparasitic Activity
Source: ACS Omega. 2026 Aug 21;11(34):50796–804. doi: 10.1021/acsomega.6c01245 (PMC13625110; doi:10.1021/acsomega.6c01245)
Supplement: Supplementary file 1 [file ao6c01245_si_001.pdf]

Supporting Information for manuscript

Isolation and structural characterization of refined proanthocyanidin fractions reveals polymerization and prodelphinidin ratio are major contributors to anti-parasitic activity

Milla M. Leppä[1], Andrew R. Williams[2], Maarit Karonen[1], Juha-Pekka Salminen[1] and Marica T. Engström[3] *

[1] Natural Chemistry Research Group, Department of Chemistry, University of Turku, Henrikinkatu 2, FI-20014 Turku, Finland
[2] Department of Veterinary and Animal Sciences, Faculty of Health and Medical Sciences, University of Copenhagen, Denmark
[3] Bioanalytical laboratory, Institute of Biomedicine, Faculty of Medicine, University of Turku, Finland
* Correspondence: mtengs@utu.fi

Table S1. The plant species and tissues used in this study. The plant sources are similar as utilized earlier [1] .

**Plant species**		Semipreparative source material	
**Latin name**	Plant tissue	[a]mDP	[b]PC/PD ratio
Aesculus hippocastanum L.	leaves	4	99/1
Trifolium medium L.	flowers	9	99/1
Rhododendron dichroanthum Diels.	leaves	5	79/21
Rhododendron schlippenbachii Maxim.	leaves	6	78/22
Larix sp	needles	12	62/38
Lotus corniculatus L.	green brownish pods	14	45/55
Lysimachia vulgaris L.	flowers	20	27/73
Pinus sylvestris L.	needles	18	24/76
Salix phylicifolia L.	leaves	20	15/85
Ribes alpinum L.	leaves	19	3/97
Trifolium repens L.	flowers	18	2/98

[b]Mean degree of polymerization (mDP) and [b]ratio of procyanidin and prodelphinidin subunits (PC/PD) measured by UPLC-MS/MS [2].

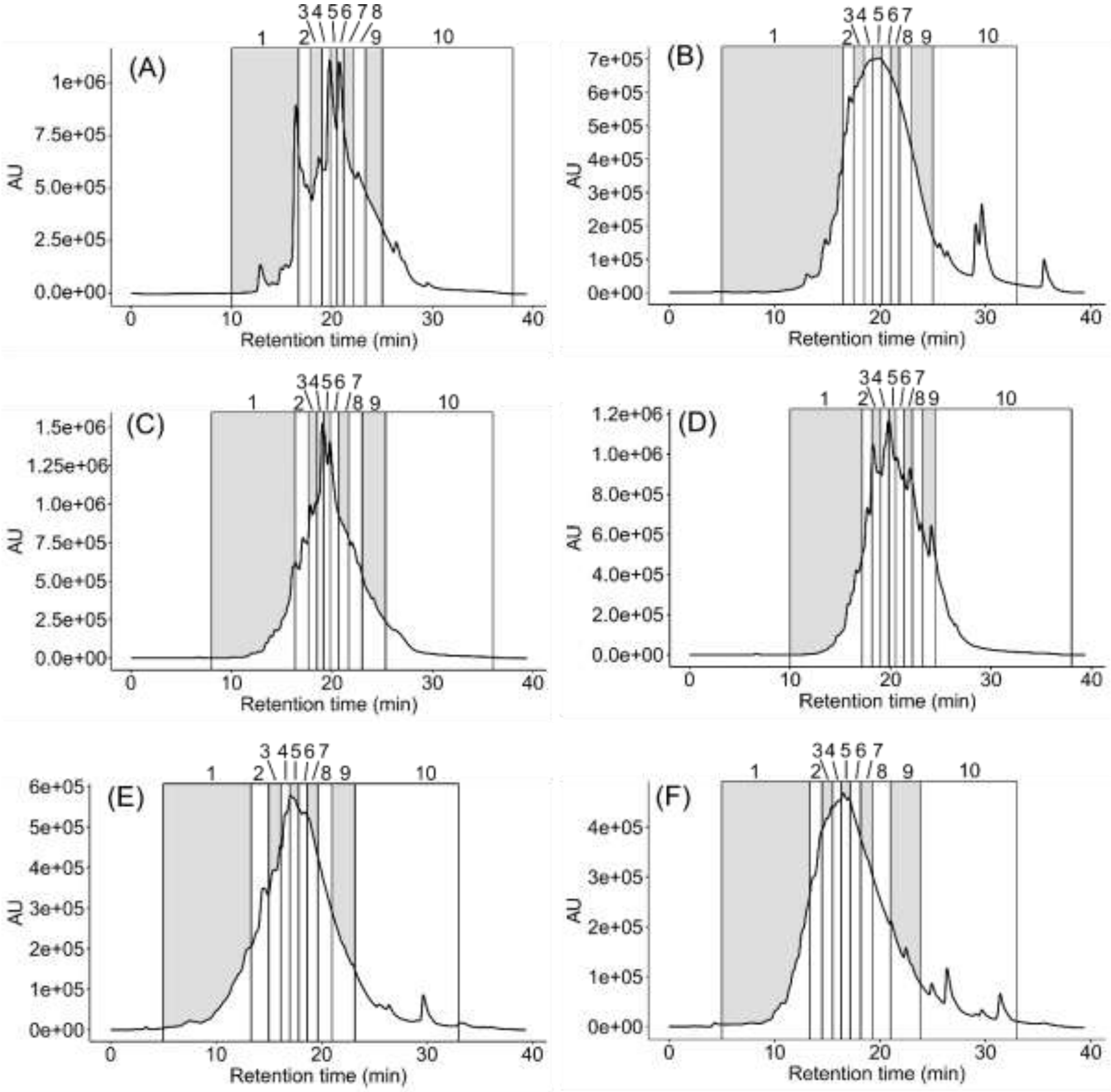

Figure S1. The UV chromatograms (λ = 280 nm) of the semipreparative purification of the pre-purified of (A) *Aesculus hippocastanum*, (B) *Trifolium medium*, (C) *Rhododendron dichroanthum*, (D) *Rhododendron schlippenbachii*, (E) *Larix* sp, (F) *Lotus corniculatus*. The pooled fractions (1–10) are presented on top of the chromatograms.

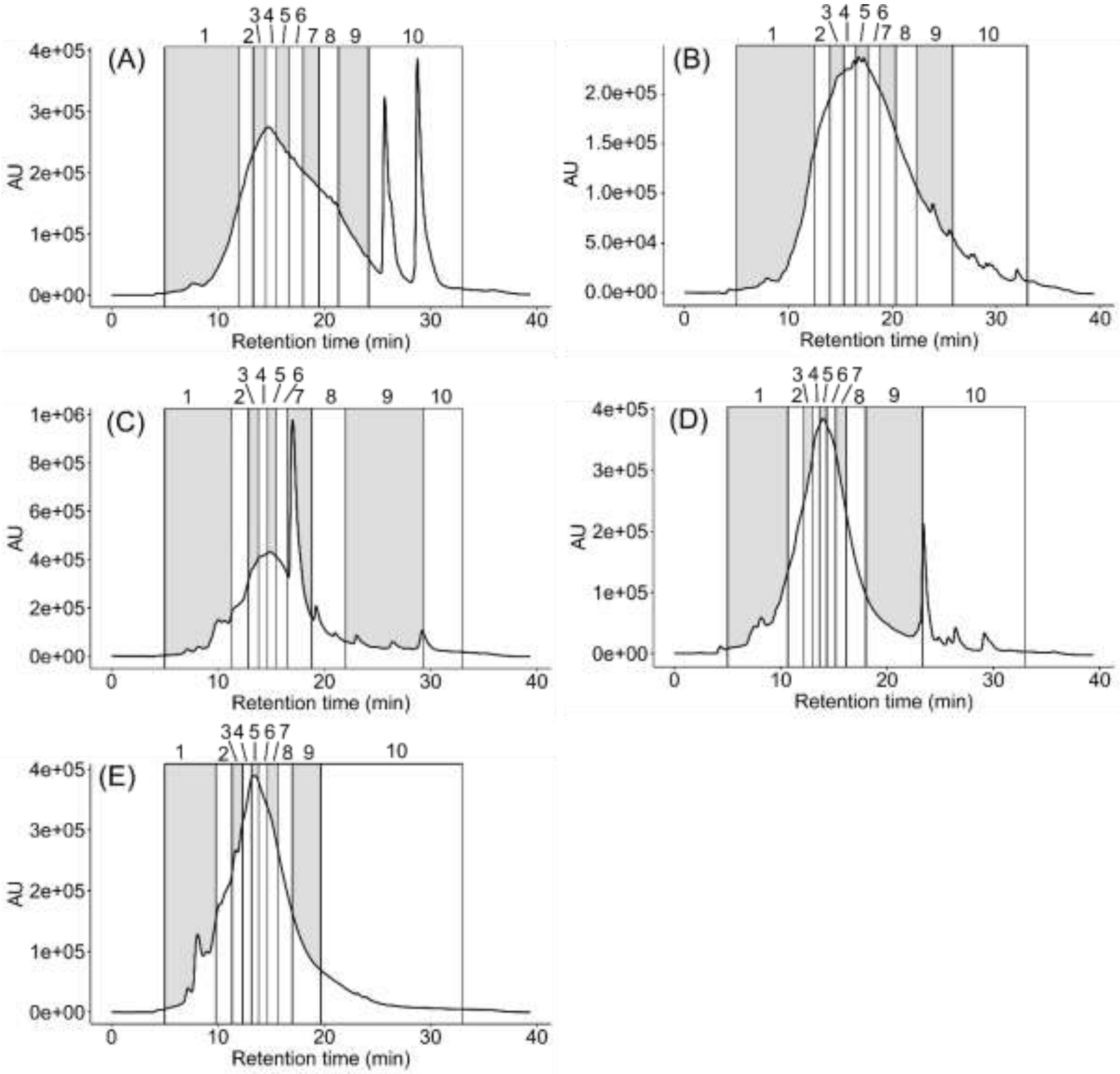

Figure S2. The UV chromatograms (λ = 280 nm) of the semipreparative purification of the pre-purified of (A) *Lysimachia vulgaris*, (B) *Pinus sylvestris*, (C) *Salix phylicofilia,* (D) *Ribes alpinum* and (E) *Trifolium repens*. The pooled fractions (1–10) are presented on top of the chromatograms.

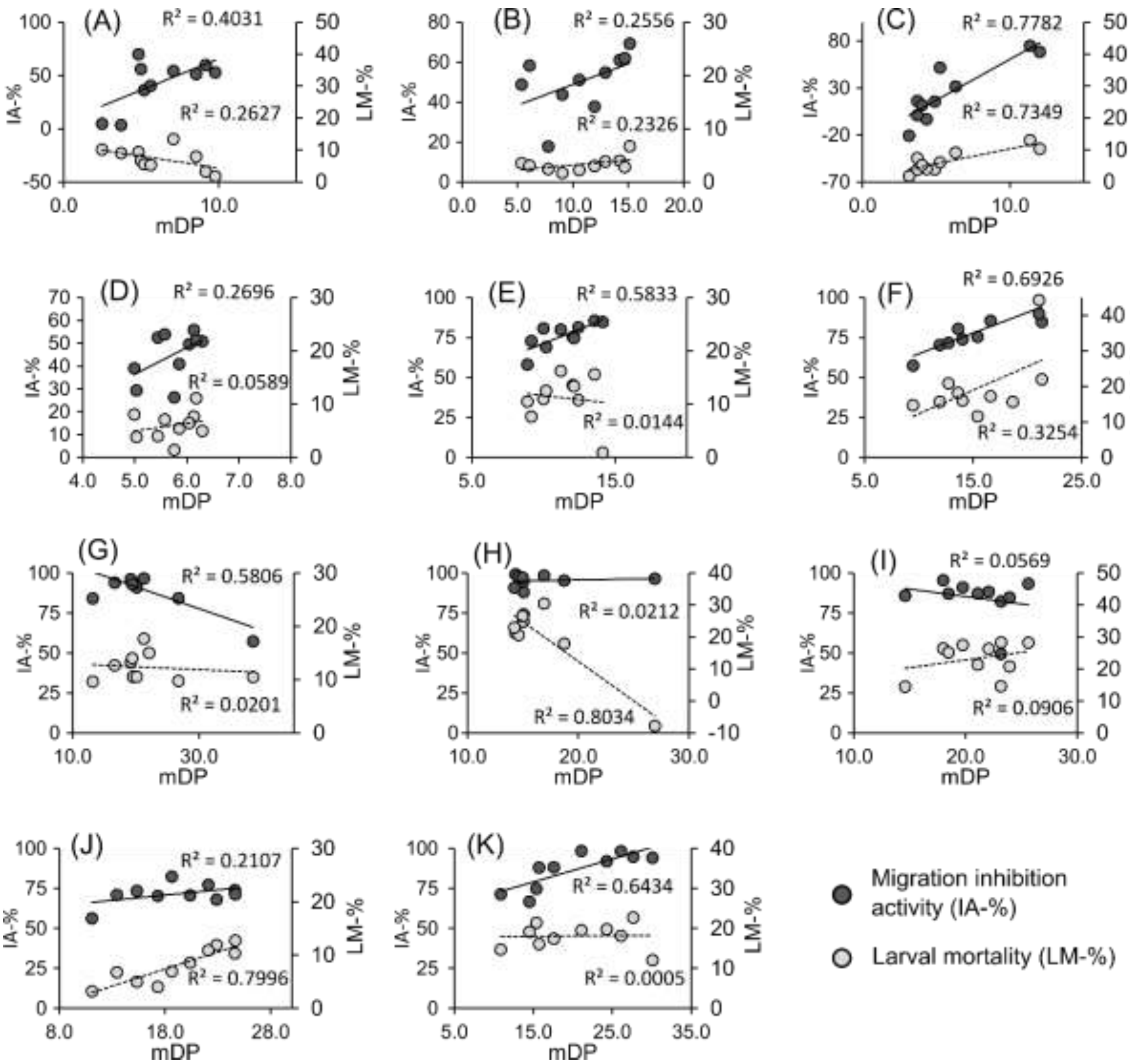

Figure S3. The migration inhibition activity (IA-%) and larval mortality (LM-%) of each studied fraction as a function of mean degree of polymerization (mDP). Dark grey: migration inhibition activity -% (IA-%), light grey: LM-%, (A) *Aesculus hippocastanum*, (B) *Trifolium medium*, (C) *Rhododendron dichroanthum*, (D) *Rhododendron schlippenbachii*, (E) *Larix* sp, (F) *Lotus corniculatus*, (G) *Lysimachia vulgaris*, (H) *Pinus sylvestris*, (I) *Salix phylicofilia*, (J) *Ribes alpinum* and (K) *Trifolium repens*.

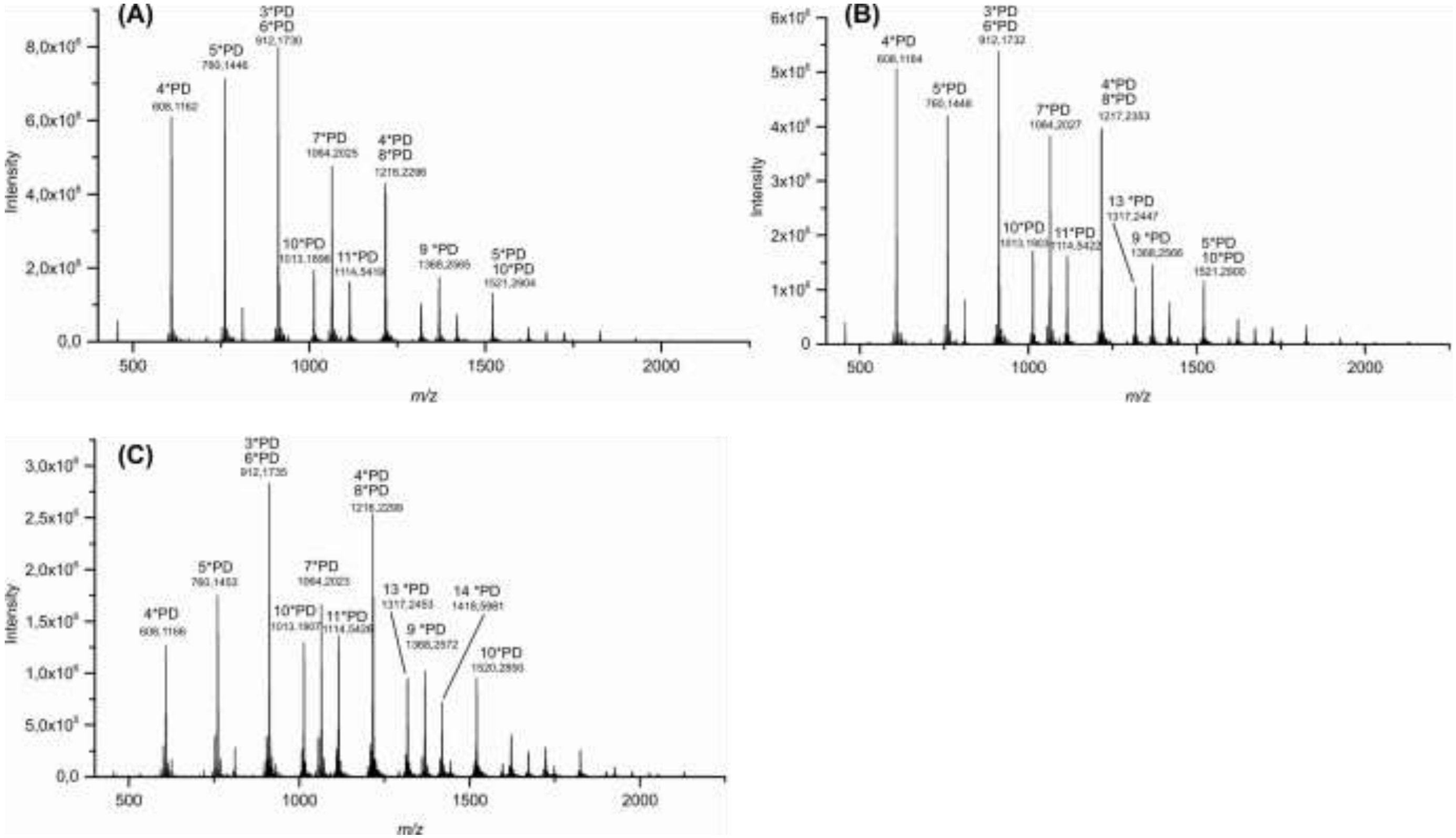

Figure S4. The high-resolution mass spectra of *T. repens* pooled semipreparative fractions (A) 2, (B) 5, and (C) 7. The interpretations on top of the mass spectral signals present proanthocyanidins consisting from a differing number of prodelphinidin (PD) units.

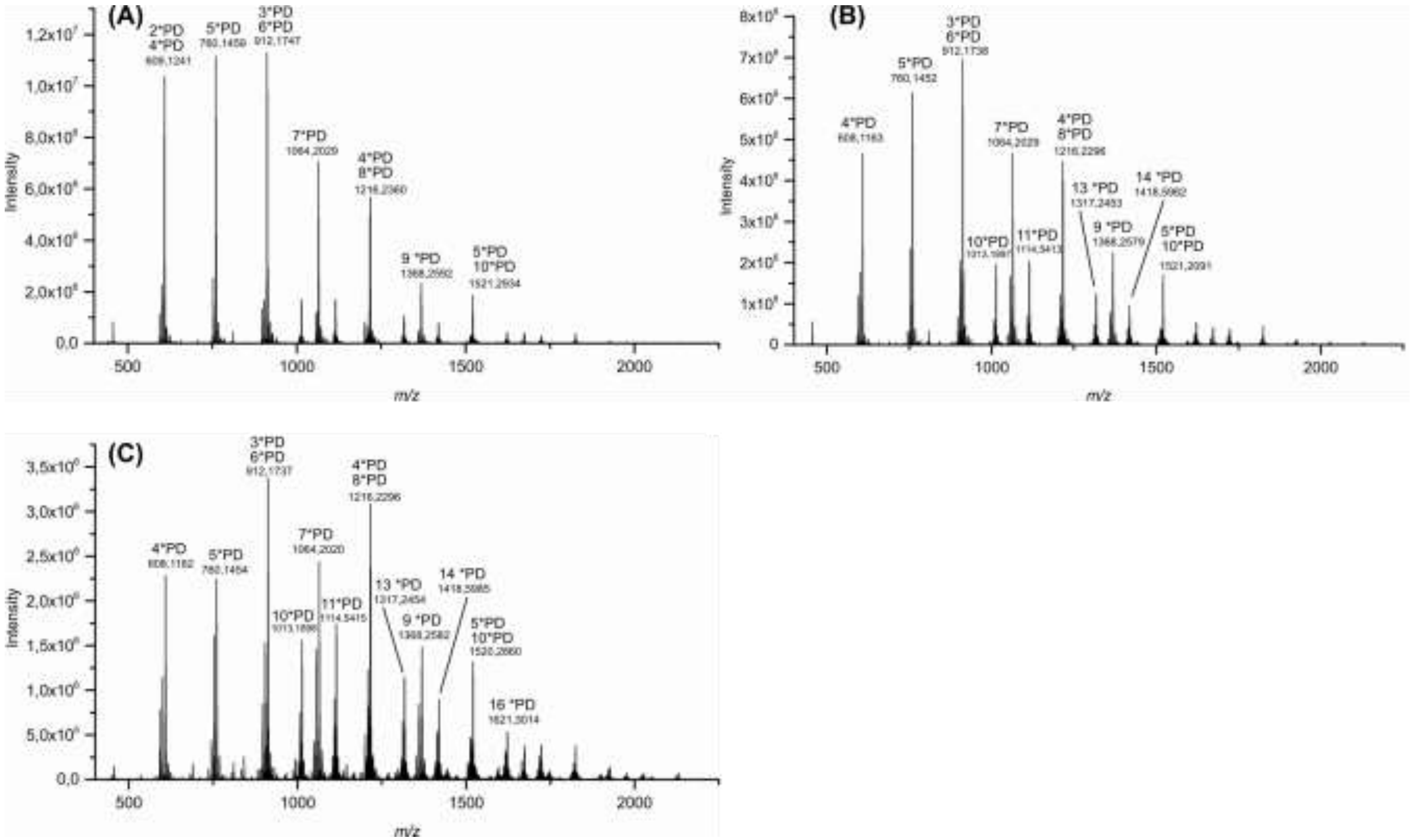

Figure S5. The high-resolution mass spectra of *R. alpinum* pooled semipreparative fractions (A) 2, (B) 5, and (C) 7. The interpretations on top of the mass spectral signals present proanthocyanidins consisting from a differing number of prodelphinidin (PD) units.

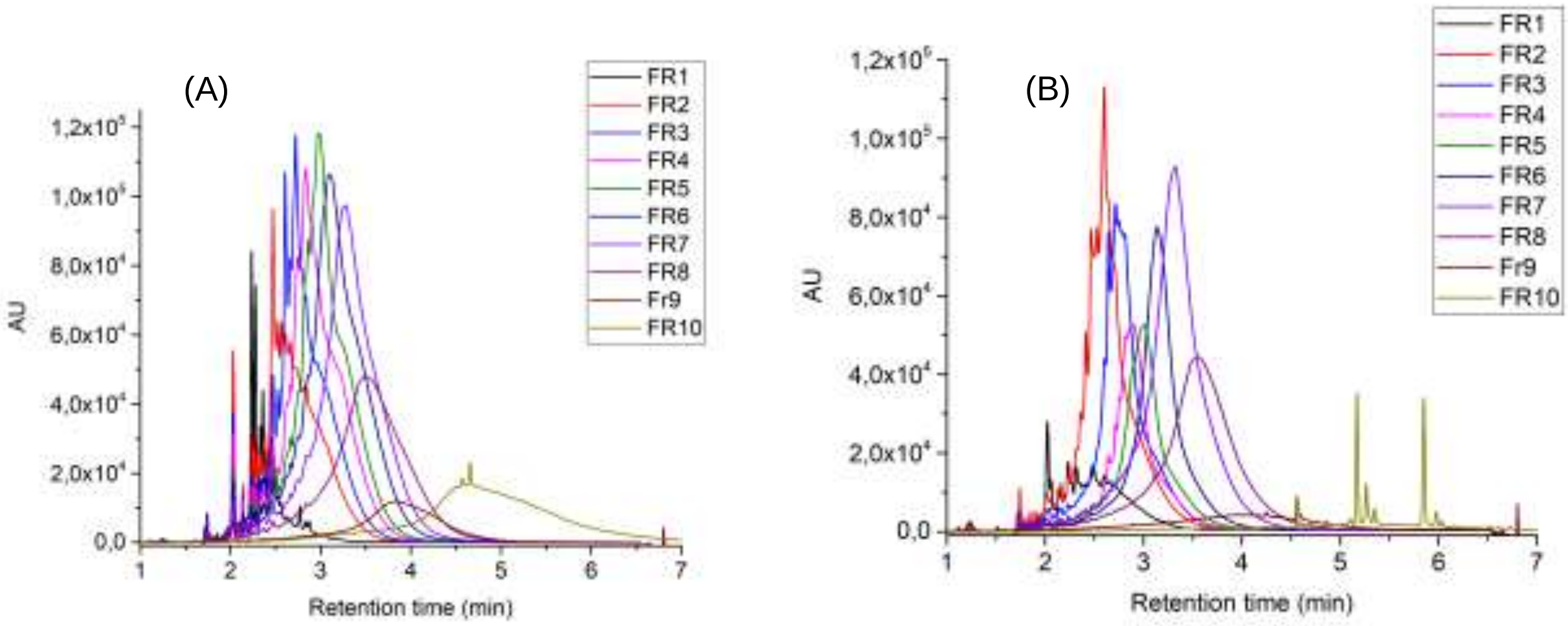

Figure S6. The UPLC UV traces (λ = 280 nm) of the pooled semipreparative HPLC fractions of (A) *R. alpinum* and (B) *T. repens*. The colors of the different fractions are presented in the legends.

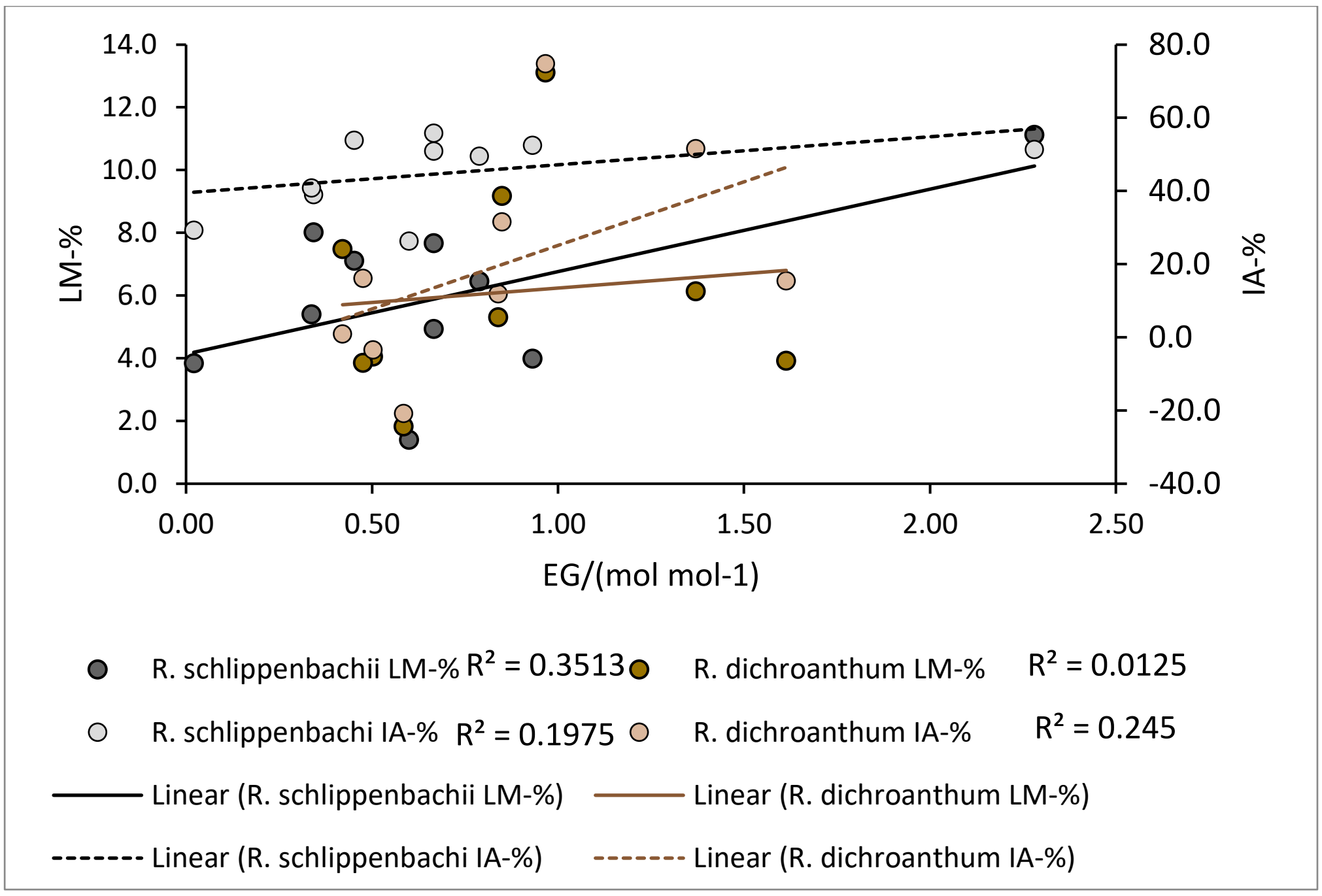

Figure S7. The larval mortality (LM-%) and migration inhibition activity (IA-%) as a function of the estimated relative galloyl content (EG) of the galloylated PAs in the plant species *R. dichroanthum* and *R. schlippenbachii*. The R^2 values are as follows: *R. dichroanthum* IA-% R^2 = 0.1975, *R. dichroanthum* LM-% R^2 = 0.3515, *R. schlippenbachii* IA-% R^2 = 0.245 and *R. schlippenbachii* LM-% R^2 = 0.0125.

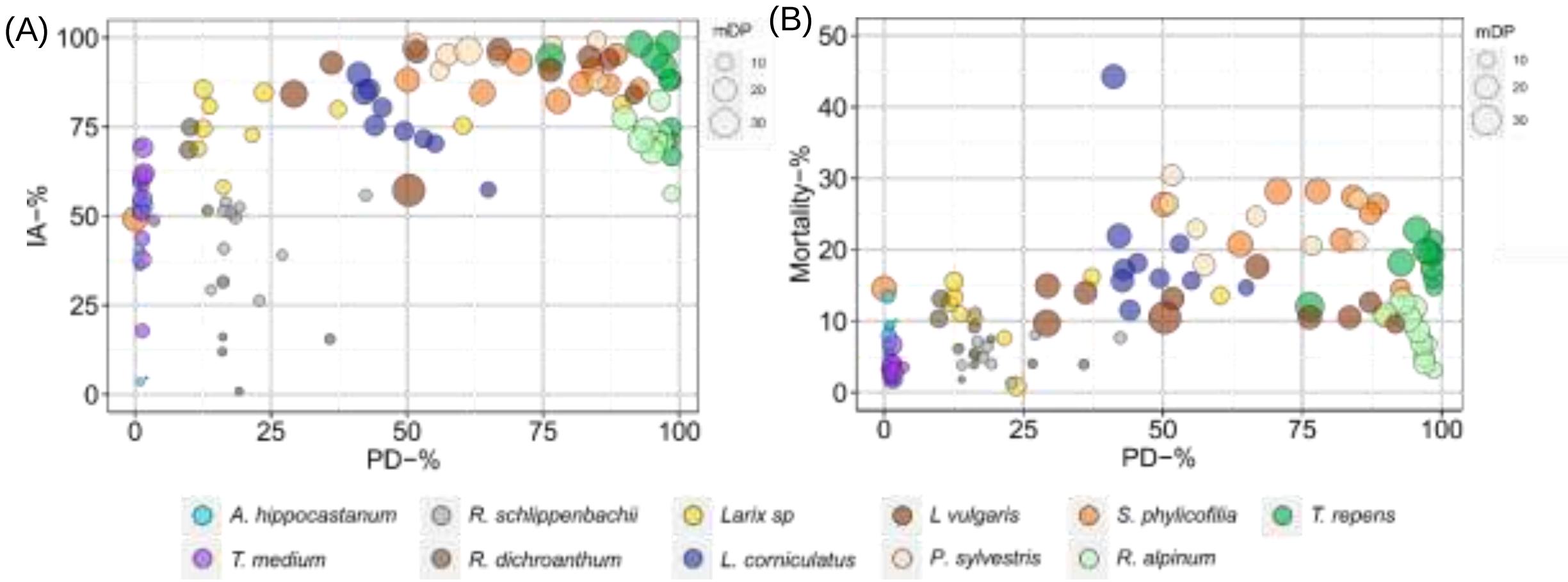

Figure S8. (A) Migration inhibition activity (IA-%) and (B) larval mortality (mortality-%) of the studied samples as a function of the proportion of prodelphinidin units (PD-%) in relation to procyanidins. The plant species are illustrated with different colors. The size of the bubbles illustrated the mean degree of polymerization (mDP) of the studied fractions.

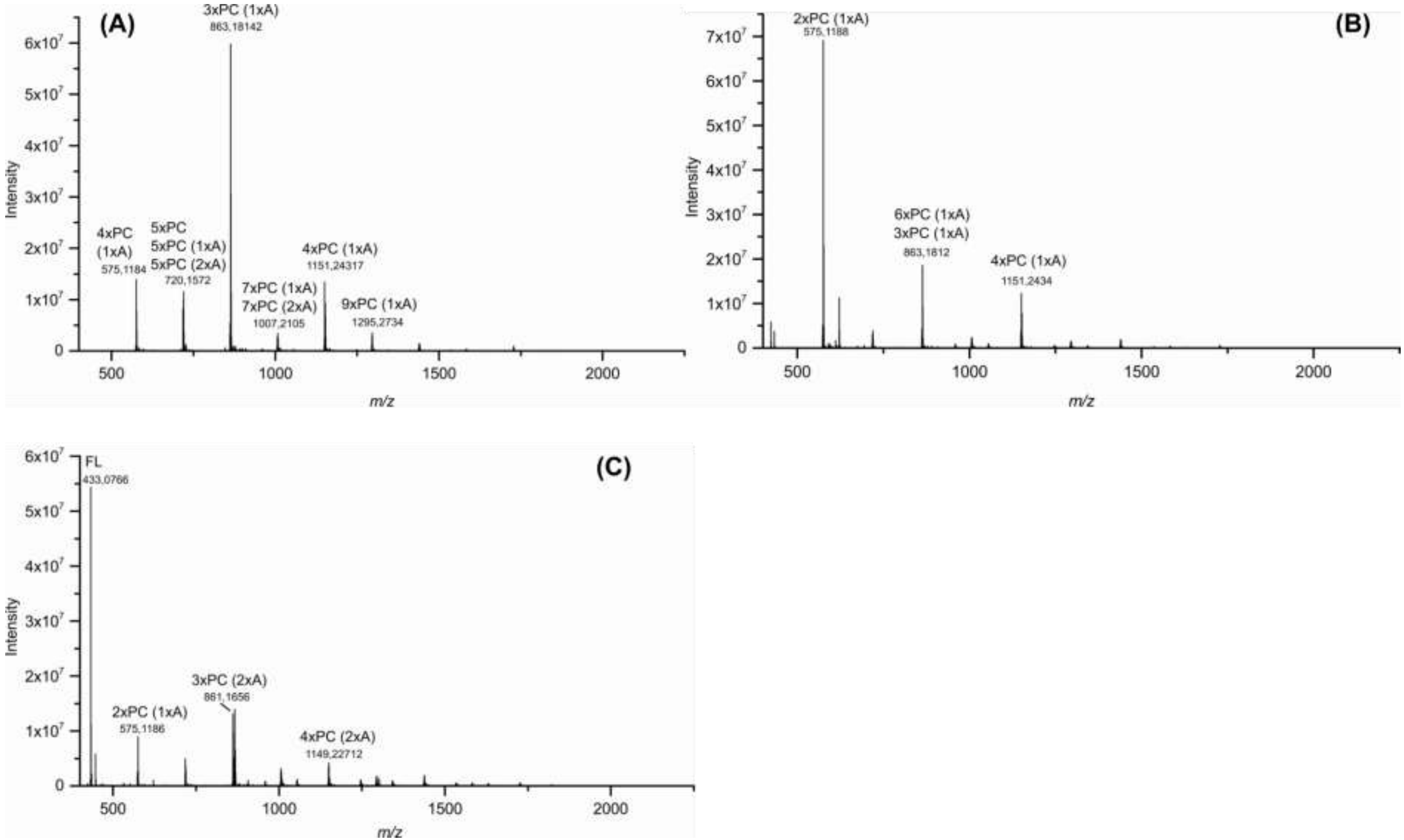

Figure S9. The high-resolution mass spectra of *A. hippocastanum* pooled semipreparative fractions (A) 2, (B) 5, and (C) 7. Interpretations on top of the mass spectral signals present proanthocyanidins consisting from a differing number of procyanidin (PC) units. In some compounds, also 1–3 A-type bonds were present and the number of the A-type bond is expressed as in the brackets as ("X"xA).

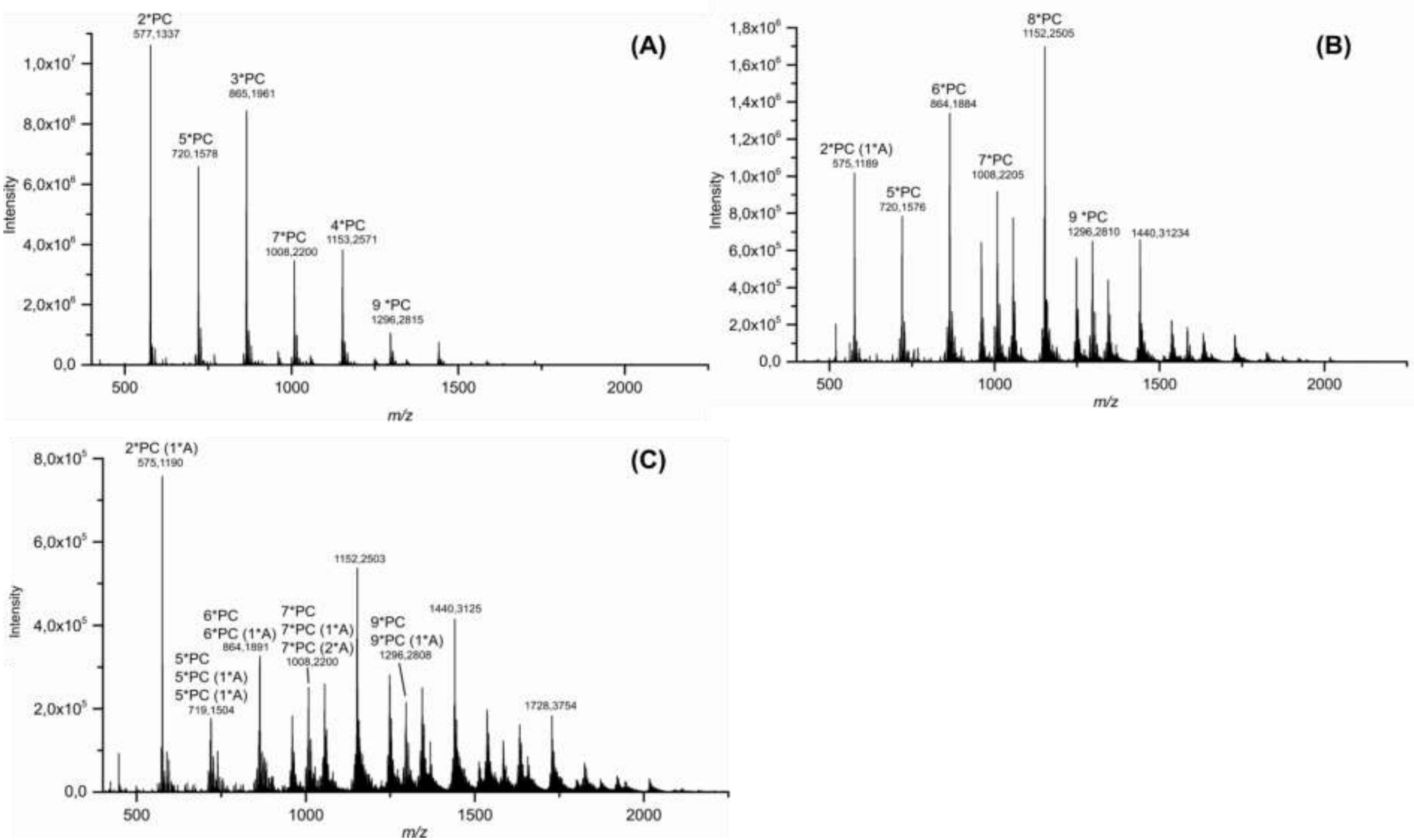

Figure S10. The high-resolution mass spectra of *T. medium* pooled semipreparative fractions (A) 2, (B) 5, and (C) 7. Interpretations on top of the mass spectral signals present proanthocyanidins consisting from a differing number of procyanidin (PC) units. In some compounds, also 1–2 A-type bonds were present and the number of the A-type bond is expressed as in the brackets as (“X”xA)

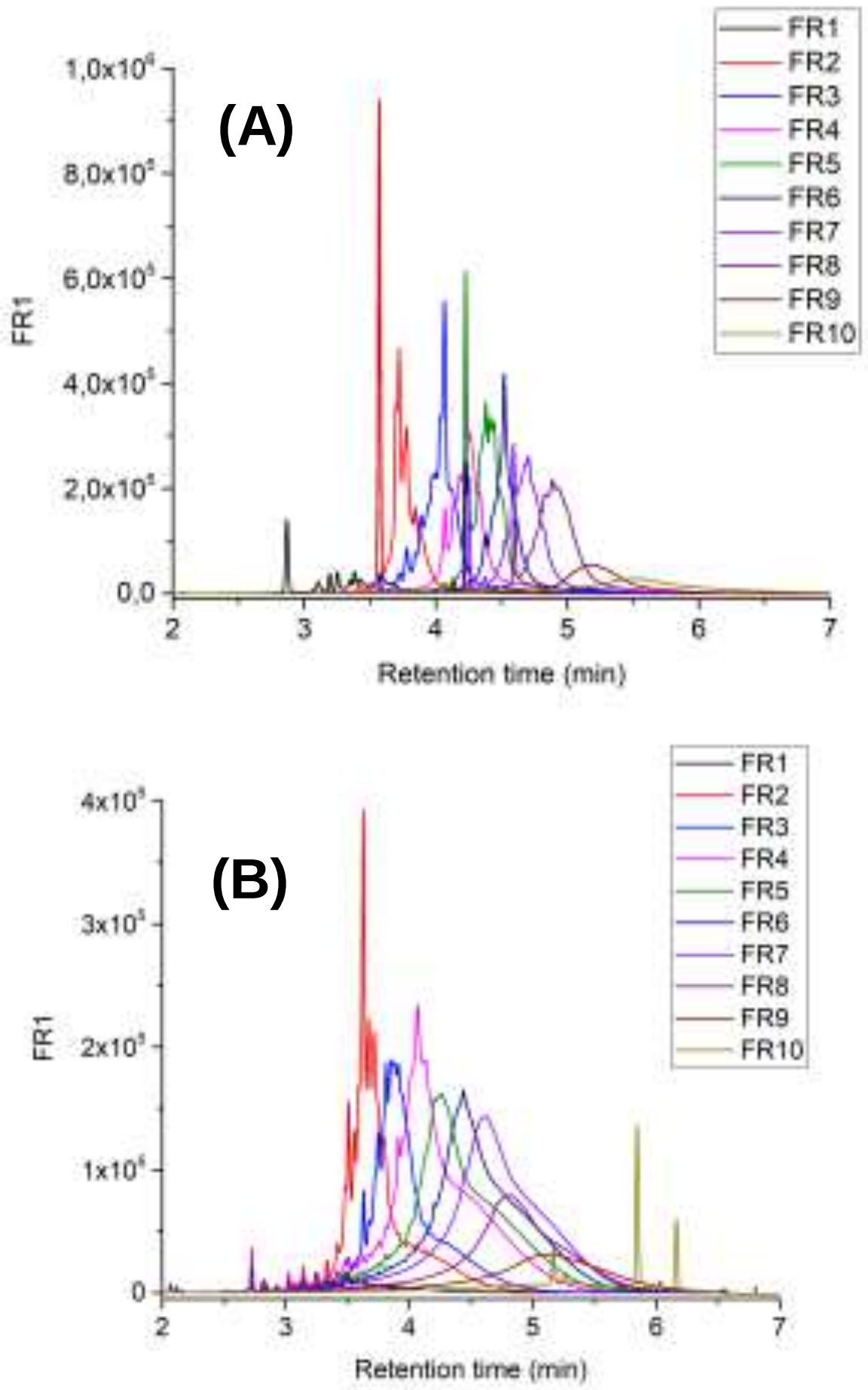

Figure S11. The UPLC DAD traces (λ = 280 nm) of the pooled semipreparative HPLC fractions of (A) *A. hippocastanum* and (B) *T. medium*. The colors of the different fractions are presented in the legends.

References

[1] Leppä, M.M.; Karonen, M.; Tähtinen, P.; Engström, M.T.; Salminen, J.-P. Isolation of chemically well-defined semipreparative liquid chromatography fractions from complex mixtures of proanthocyanidin oligomers and polymers. J. Chromatogr. A 2018, 1576, 67–79. https://doi.org/10.1016/j.chroma.2018.09.034

[2] Engström, M.T.; Pälijärvi, M.; Fryganas, C.; Grabber, J.H.; Mueller-Harvey, I.; Salminen, J.-P. Rapid qualitative and quantitative analyses of proanthocyanidin oligomers and polymers by UPLC-MS/MS. J. Agric. Food Chem. 2014, 62, 3390–3399. https://doi.org/10.1021/jf500745y
